# A Case of Achalasia Presented with Cardiopulmonary Arrest

**DOI:** 10.1155/2012/794858

**Published:** 2012-12-24

**Authors:** Fatih Altintoprak, Bumin Degirmenci, Enis Dikicier, Guner Cakmak, Taner Kivilcim, Omer Yalkin, Gokhan Akbulut, Osman Nuri Dilek

**Affiliations:** ^1^Department of General Surgery, Faculty of Medicine, Sakarya University, 54100 Sakarya, Turkey; ^2^Department of Radiology, Faculty of Medicine, Sakarya University, 54100 Sakarya, Turkey; ^3^Department of General Surgery, Research and Educational Hospital, Sakarya University, 54100 Sakarya, Turkey

## Abstract

Achalasia is a rare disorder characterised by obstruction of the distal oesophagus and subsequent dilation of the proximal oesophagus. Patients generally complain of gastrointestinal symptoms; however, pulmonary symptoms and complications may also occur. A 35-year-old woman was brought to our emergency service complaining of sudden-onset dyspnea that started 15 minutes earlier during dinner. She suffered a cardiopulmonary arrest due to aspiration 5 minutes after being admitted to the emergency room and was intubated. Thoracic computed tomography examination showed that her oesophagus was filled with undigested food. Heller cardiomyotomy and Dor fundoplication was performed via laparotomy with the diagnosis of primary achalasia, and she was discharged as uneventful on the 5th postoperative day.

## 1. Introduction

Achalasia is a rare motor disorder of the oesophagus and lower oesophageal sphincter. Primary achalasia is rare, with an annual incidence of 1/100,000 individuals [1]. Secondary achalasia has the same clinical findings as primary achalasia, but the aetiology can be identified. Pseudoachalasia arises as a result of infiltration of the lower oesophageal sphincter by malignancy or amyloidosis. The most common complaints in achalasia are progressive dysphagia, chest pain, regurgitation, and weight loss [2]. Over the years, patients who are not treated develop a dilated oesophagus. In rare cases, the patients' first complaints may be related to the respiratory system [3]. Here, we present our treatment approach to achalasia diagnosed after a cardiopulmonary arrest that followed aspiration. We later learned that the patient had a history of dysphagia. We also review the pertinent literature. 

## 2. Case Report

**Figure 1:**
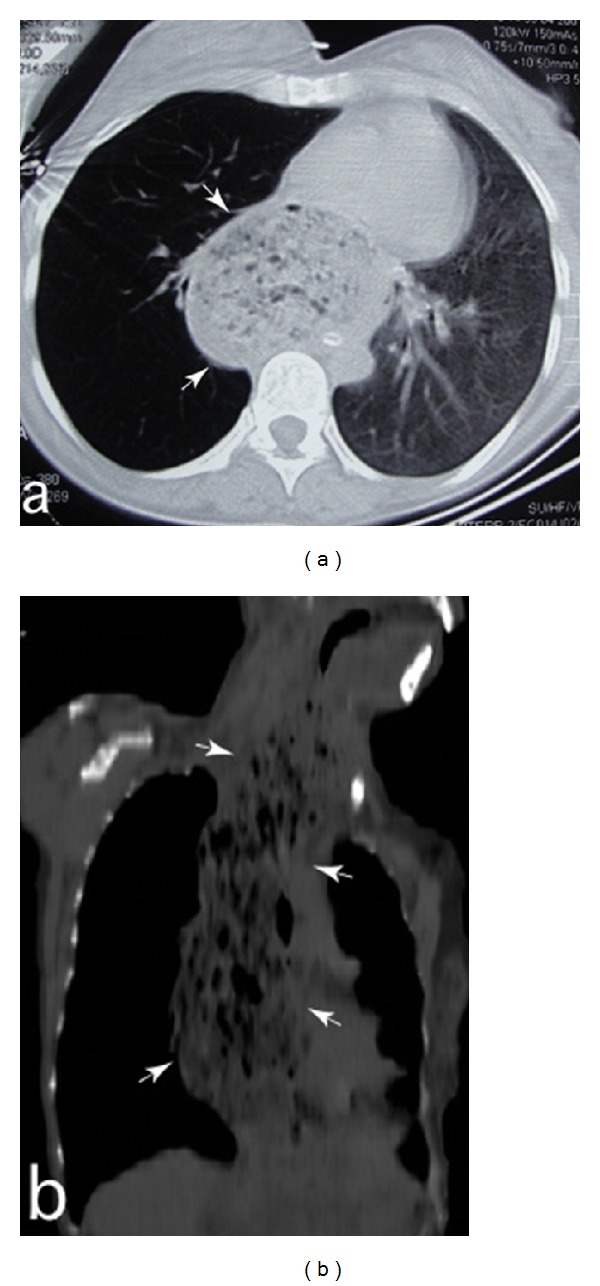
Thoracic computed tomography examination after intubation. (a) The axial cross-section shows a severely dilated oesophagus filled with food residue behind the trachea (arrows). The left lung parenchyma was infiltrative due to aspiration, and the heart was compressed by the dilated esophagus. (b) The coronal cross-section shows that the oesophagus is filled with food and air up to the cervical region (arrows).

**Figure 2:**
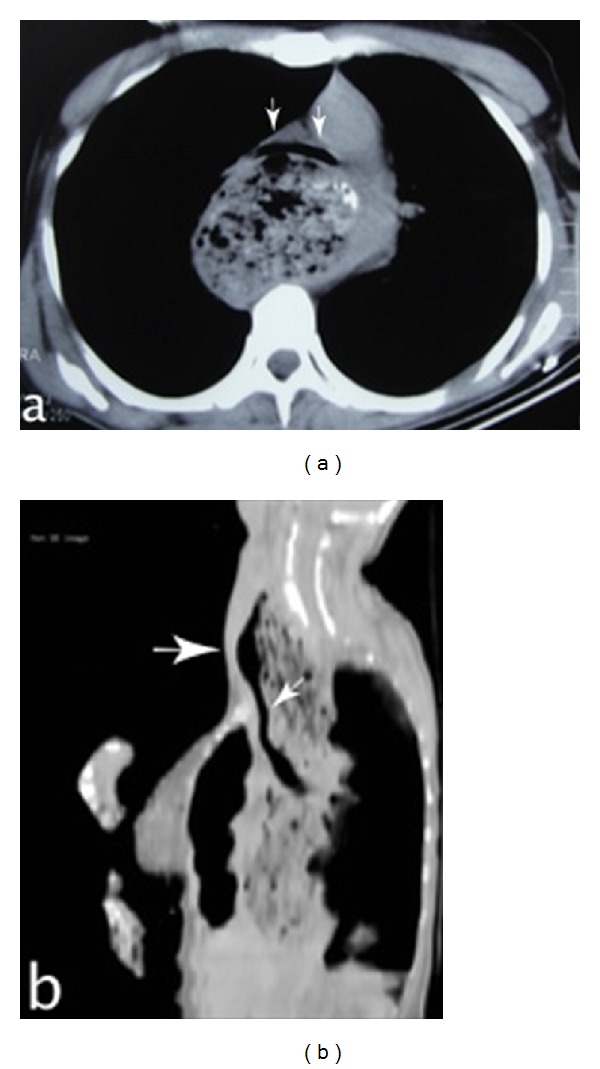
(a) Axial cross-section shows stenosis resulting from the pressure of the oesophagus on the trachea at the carina level (arrows). (b) Sagittal cross-section shows that the diameter of the proximal trachea is normal (large arrow), and stenosis of the lumen is present due to the pressure on the distal trachea (small arrow).

A 35-year-old woman was brought to our emergency service with the complaining of sudden onset respiratory distress. Her symptoms has started 15 minutes earlier, with acute onset dysphagia and increased dyspnea during dinner. She had no history of respiratory or cardiac disease. On physical examination, she appeared cyanotic and anxious and had audible expiratory wheezing. Her pulse was 112 bpm, blood pressure was 90/50 mm Hg, and respiratory rate was 32–36 rpm. Her arterial blood gases were pH 7.52, pCO_2_ 24.7, and pO_2_ 72.4 mm Hg, and the bicarbonate concentration was 19.2 mmol/L. Her electrocardiogram showed sinus tachycardia. Intravenous (IV) fluid resuscitation (0.9% NaCl) and oxygen via a nasal cannula (4 L/min) were started immediately. The patient was intubated owing to cardiopulmonary arrest five minutes after starting treatment and was resuscitated (external heart massage, IV adrenalin and atropine administration) during seven minutes. In course of resuscitation, 15–20 mL of brown fluid were aspirated through the endotracheal tube. Thoracic computed tomography (CT) while the patient was intubated showed that the oesophagus was wide enough to fill the mediastinum, and it was filled with food residue; the left lung parenchyma was infiltrated, in accordance with aspiration, and the heart was compressed by the dilated esophagus (Figure 1). Stenosis of the distal trachea and main bronchi was observed in the coronal and axial cross-sections due to the pressure exerted by the oesophagus (Figure 2). A nasooesophageal tube was placed to drain the oesophagus. The patient was admitted to intensive care; 24 hours after being extubated, she stated that she had had difficulty swallowing for 18 years. An endoscopic assessment of the upper gastrointestinal system was unsuccessful because of the food in the oesophagus. The oesophagus was drained using daily lavage with 5 L of 0.9% NaCl through the nasooesophageal tube for 3 days. A barium esophagogram taken on day 4 showed that it was dilated and severe stenosis was present at the oesophagogastric junction (bird-beak sign). Repeated thoracoabdominal CT and upper gastrointestinal endoscopy showed no evidence of pseudoachalasia, and the oesophagus and gastric mucosa were normal. Esophageal manometry showed that increased LES resting pressure (32 mm Hg), impaired LES relaxation, but no significant pressurization within the esophageal body. The patient was diagnosed with primary achalasia based on her history and diagnostic procedures results. We prefer surgical treatment in our case that lacks contradictions to surgery, and Heller cardiomyotomy and Dor fundoplication operation was performed via laparotomy. A follow-up study of the oesophagus 4 days after surgery showed that its diameter was smaller and that the contrast transition to the stomach was normal. The patient was discharged uneventfully 5 days after surgery. At the 18-month follow-up examination, the patient had no gastrointestinal or respiratory complaint.

## 3. Discussion

Although achalasia commonly presents with frequent dysphagia and weight loss, the symptoms can differ. Rarely, the presenting complaints are respiratory, including loss of voice, chronic coughing, wheezing, recurring lung infections, pneumonia, atelectasis, and breathing difficulties [4]. Regurgitation and aspiration cause the respiratory symptoms. Stridor, which is even rarer, develops as a result of tracheal compression and can be fatal if not treated. The exact mechanism of airway obstruction has not been elucidated, but several mechanisms have been suggested, including the pinch-cock valve effect, air drawn into the oesophagus secondary to negative intrathoracic pressure, upper oesophageal sphincter failure, and the loss of the normal belch reflex [5]. Additionally, arrhythmia due to excessive pressure caused by the Valsalva maneuver or underlying unknown diseases may be responsible for sudden death in achalasia patients [6, 7]. 

In our case, the patient had severe respiratory compromise and wheezing at admission. Because these symptoms developed suddenly, the patient had no respiratory complaint before admission, and fluid was aspirated from the trachea, it was thought that the patient had aspirated. We think that the aspiration may have a triggered factor for the development of cardiopulmonary arrest due to presence of stenosis of the trachea and the heart compression. 

Achalasia can present with respiratory complaints in every age group, from young children to the elderly. Usually, these patients have already been diagnosed with achalasia or the current respiratory complaints are recurring [5, 8, 9]. No case has been reported of a patient with undiagnosed achalasia admitted with acute airway obstruction with rapid deterioration and cardiopulmonary arrest. Patients admitted with acute airway obstruction usually have tracheal obstruction, which distinguishes them from our case [3, 5, 8].

Various treatments for achalasia have been described, including medical, endoscopic, and surgical methods. The effectiveness of medical treatment is extremely limited, and it is only preferred in patients for whom other treatment options are not available or patients with refractory chest pains [1]. Botulinum toxin injection is an endoscopic treatment option that is easy to perform and readily tolerated by patients. However, its duration of effectiveness is extremely variable, and more than one injection is required [1]. Balloon dilation is the most successful nonsurgical treatment; the average rate of oesophageal perforation, the most feared complication, is 2.3% (range, 0–6.6%) [10, 11]. 

The standard surgical treatment for achalasia is the Heller myotomy, although different techniques can be used. The advantages of surgical treatment include a significant reduction in symptoms, shortened hospital stay, low rate of postoperative complications, and low rate of gastro-oesophageal reflux [12]. The advantages of minimally invasive procedures (less postoperative pain, early mobilization, etc.) are well known in modern surgical practice. Although minimally invasive techniques have become the gold standard procedure for surgical treatment of achalasia, these techniques require experience. Moreover, Lopes et al. [13] reported that either approach (laparotomy or laparoscopy) can be chosen, depending on the surgeon's experiences due to the fact that in achieving the final results (relief of dysphagia) are similar. We have chosen laparotomy for myotomy. 

According to the literature, achalasia patients admitted with acute respiratory complaints generally require endotracheal intubation as an emergency treatment. Nasooesophageal decompression, defined by Dunlop and Travis [3] “as an important manoeuvre that restricts mortality” eases the patients' symptoms [8, 9, 14, 15]. A Heller myotomy is extremely effective in reducing respiratory complaints as a definitive treatment and prevents recurrent episodes in 82% of patients with recurring pneumonia [16]. However, different treatment approaches were used in patients with achalasia presenting with respiratory symptoms. While some authors prefer the surgical approach [8, 17], others treated patients with botulinum toxin [9] or balloon dilation after botulinum toxin injections [18]. Undoubtedly, patient age, comorbidity, and physician's experience play important roles in these preferences. 

 In our patient, we applied the endotracheal intubation and nasooesophageal decompression, respectively. After the diagnostic procedures, we have prefered the surgical myotomy for definitive treatment. However, this choice should not be perceived as a suggestion and should be noted that the method of treatment may be different for each patient.

In conclusion, clinicians can rarely encounter achalasia that presents acutely, with potentially fatal respiratory complaints. We believe that the main steps in the treatment algorithm (opening the airway, emergency radiological examination, draining the oesophagus, extensive radiologic, endoscopic, manometric examinations, and deciding the definitive treatment option) should be known by all clinicians.

## References

[1] Dughera L, Chiaverina M, Cacciotella L, Cisarò F (2011). Management of achalasia. *Clinical and Experimental Gastroenterology*.

[2] Reynolds JC, Parkman HP (1989). Achalasia. *Gastroenterology Clinics of North America*.

[3] Dunlop SP, Travis SPL (1997). Achalasia presenting as acute stridor. *European Journal of Gastroenterology and Hepatology*.

[4] Chapman S, Weller PH, Campbell CA, Buick RG (1989). Tracheal compression caused by achalasia. *Pediatric Pulmonology*.

[5] Doshi AH, Aw J, Costa F, Cohen L, Som PM (2009). Cervical tracheal compression in a patient with achalasia: an uncommon event. *American Journal of Neuroradiology*.

[6] Schalinski S, Guddat SS, Tsokos M, Byard RW (2009). Megaesophagus and possible mechanisms of sudden death. *Journal of Forensic Sciences*.

[7] Sperry K (1994). Achalasia, the valsalva maneuver, and sudden death: a case report. *Journal of Forensic Sciences*.

[8] Healy JS, Baillie J (2007). Acute airway obstruction as a complication of untreated achalasia. *Endoscopy*.

[9] Arcos E, Medina C, Mearin F, Larish J, Guarner L, Malagelada JR (2000). Achalasia presenting as acute airway obstruction. *Digestive Diseases and Sciences*.

[10] Novais PA, Lemme EMO (2010). 24-h pH monitoring patterns and clinical response after achalasia treatment with pneumatic dilation or laparoscopic Heller myotomy. *Alimentary Pharmacology and Therapeutics*.

[11] Kadakia SC, Wong RKH (2001). Pneumatic balloon dilation for esophageal achalasia. *Gastrointestinal Endoscopy Clinics of North America*.

[12] Litle VR (2008). Laparoscopic Heller myotomy for achalasia: a review of the controversies. *Annals of Thoracic Surgery*.

[13] Lopes LR, Braga Nda S, Oliveira GC, Coelho Neto Jde S, Camargo MA, Andreollo NA (2011). Results of the surgical treatment of non-advanced megaesophagus using Heller-Pinotti's surgery: Laparotomy vs. Laparoscopy. *Clinics*.

[14] Khan AA, Shah SWH, Alam A (2007). Achalasia Esophagus; presenting as acute air way obstruction. *Journal of the Pakistan Medical Association*.

[15] Wagh MS, Matloff DS, Carr-Locke DL (2004). Life-threatening acute airway obstruction in achalasia. *MedGenMed*.

[16] Khandelwal S, Petersen R, Tatum R (2011). Improvement of respiratory symptoms following Heller myotomy for achalasia. *Journal of Gastrointestinal Surgery*.

[17] Bacellar P, Silva M, Tinoco N, Costa F (2008). Oesophagus achalasia: differencial diagnosis of asthma. *Revista Portuguesa de Pneumologia*.

[18] Basavarajaiah S, Barnes DJ, Boyle N (2011). Simple treatment of stridor caused by achalasia of the cardia. *Journal of the Royal Society of Medicine*.

